# Headspace Extraction onto a 3D-Printed Device for GC-MS Quantification of Polychlorinated Biphenyls in Newborn Urine

**DOI:** 10.3390/ijms26062755

**Published:** 2025-03-19

**Authors:** Paweł Georgiev, Mariusz Belka, Szymon Ulenberg, Dagmara Kroll, Bartosz Marciniak, Izabela Drążkowska, Tomasz Bączek, Justyna Płotka-Wasylka

**Affiliations:** 1 Department of Pharmaceutical Chemistry, Medical University of Gdańsk, J. Hallera 107, 80-416 Gdańsk, Poland; pawel.georgiev@gumed.edu.pl (P.G.);; 2 Division of Neonatology, Medical University of Gdańsk, 80-210 Gdańsk, Poland; 3 Department of Analytical Chemistry, Faculty of Chemistry, Gdańsk University of Technology, G. Narutowicza 11/12, 80-233 Gdańsk, Poland; juswasyl@pg.edu.pl; 4 BioTechMed Center, Gdańsk University of Technology, G. Narutowicza 11/12, 80-233 Gdańsk, Poland

**Keywords:** 3D printing, headspace extraction, environmental analysis, digital light processing (DLP), silica particles, sorption, desorption

## Abstract

Polychlorinated biphenyls (PCBs) are persistent organic pollutants that pose significant health risks, especially for neonates. Traditional urine analysis methods for PCBs are often complex and prone to contamination. This study introduces a novel, efficient, and contamination-free method for PCB analysis in neonatal urine using 3D-printed extraction devices. A headspace extraction method was developed, utilizing a 3D-printed device containing C18-modified silica particles. Urine samples were heated to 90 °C, and volatile PCBs were sorbed onto the particles. The method was optimized for maximum extraction efficiency and selectivity, demonstrating excellent linearity, precision, and accuracy. The optimized method was successfully applied to analyze neonatal urine samples, revealing detectable levels of PCBs. This innovative approach, leveraging 3D-printed devices, offers a promising solution for sample preparation, minimizing contamination risks and enabling the analysis of volatile compounds. The customizable nature of 3D-printed devices opens up possibilities for future advancements in environmental analysis.

## 1. Introduction

Polychlorinated biphenyls (PCBs) were produced from the 1930s until the 1970s [1]. Their high combustion temperature attracted industrial interest for use as flame retardants (in the furniture industry), electrical insulators, and hydraulic fluids in capacitors and transformers [2]. After approximately 1.5 billion pounds of PCBs were produced in the USA, it became evident that they were highly toxic and very persistent, leading to their accumulation in the environment [3]. It was not until 1979 that PCBs were finally banned after their carcinogenic effects and ability to accumulate in the environment were publicly disclosed [4]. Testing PCB levels in children’s urine is extremely important due to the potential health risks posed by these chemical compounds. PCBs are synthetic substances that can bioaccumulate in living organisms, including human tissues [5,6]. The majority of PCB intake in humans comes from food (about 97%), with much smaller amounts from air and water [7]. The most contaminated sources are materials and products of marine origin, especially fatty fish and seafood [8].

PCBs are a group of synthetic organic chemicals with various chemical structures [9,10]. They are characterized by a biphenyl structure, which consists of two benzene rings connected by a single bond with chlorine atoms attached in various positions to the benzene rings [11]. There are 209 different congeners of PCBs, each with its own unique chemical structure [12]. The specific arrangement of chlorine atoms determines the properties of each compound, including toxicity [13]. PCBs are highly persistent in the environment and can bioaccumulate in organisms through the food web [14].

Short-term exposure to PCBs can have immediate and acute health effects, including skin rash, itching, eye irritation, respiratory issues, gastrointestinal problems, nausea, vomiting, and diarrhea [15]. The health effects of long-term exposure to PCBs include various types of cancer, such as liver, breast, and skin cancer [16]. PCB exposure during pregnancy can lead to developmental problems in the fetus, such as low birth weight, cognitive deficits, and impaired motor skills [17]. It is crucial to mitigate exposure to PCBs to prevent these health risks. For this purpose, it is necessary to develop new and sensitive analytical methods for determining PCBs in biological samples coming from newborns and children.

In the literature, there are methods for determining PCBs in urine and other aqueous samples. Most commonly encountered is liquid–liquid extraction (LLE) [18,19,20,21]. This method utilizes relatively large amounts of strong organic solvents, which is not in line with the principles of green chemistry [22]. LLE often lacks the selectivity needed for PCB extraction, leading to co-extraction of other undesired compounds. This can complicate subsequent analysis and decrease quantitative accuracy [23]. On the other hand, another method used for PCB extraction is solid-phase extraction (SPE) [24,25,26]. While SPE offers improved selectivity, compared to LLE, it typically involves multiple steps, which can prolong the extraction process and introduce potential sources of error [27].

A promising alternative to the above-mentioned methods is the use of extraction devices obtained by application of 3D printing [28,29]. Three-dimensional printing presents several advantages, such as minimized waste production (as it is possible to print the exact number of extraction devices), which is consistent with the principles of green chemistry. In addition, 3D printing makes it easier to optimize the method depending on the sample size or analytical laboratory equipment [30]. Three-dimensional printers are cheap to purchase and maintain, which makes them a solid alternative in sample preparation [29]. The use of printed extraction devices allows thermal sorption without the use of an organic solvent, which makes the sample preparation procedure simple, fast, and safe for the person performing the experiment. Furthermore, the presented alternative of headspace sorption is more selective, enabling the extraction of only volatile compounds that have an affinity towards used sorbent particles and minimizing the extraction of undesirable chemical compounds that may be present in the liquid sample. This enables obtaining clean extracts, facilitating the analysis and interpretation of results.

This study presents the development of a GC-MS procedure for the quantification of selected PCBs in newborn urine. The presented research is a continuation of studies on the development of an extraction device using DLP (digital light processing) 3D printing technology employing porous silica suspension as a raw material [31]. In-lab fabricated sorbent devices were employed, consisting of acrylate resin cured with a 3D printer and incorporating C18-modified silica particles. We present the development of the experimental setup and optimization of extraction parameters. The applicability of the developed extraction method using 3D-printed extraction devices was validated through the analysis of newborn urine samples. This innovative approach offers a promising alternative for sample preparation by utilizing and customizing 3D-printed extraction devices, offering customizable size, shape, and chemical properties.

## 2. Results and Discussion

### 2.1. Primary Assessment of Device Performance and Temperature Effects

**Figure 1:**
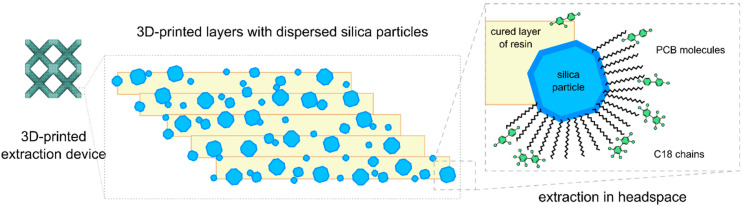
Overall design of the extraction device and mechanism PCBs retention on C18-modified silica particles.

The primary objective of this study was to evaluate the applicability of a 3D-printed device for the extraction of PCBs from urine coming from newborns. The fabrication of this device via 3D printing has been previously reported [32]. The extraction sorbent consists of C18-modified silica particles embedded within a commercial acrylate-based resin matrix. Previous work demonstrated that minimizing the bulk volume of the 3D-printed sorbent enhances both extraction efficiency and kinetics. Consequently, a volumetric lattice structure with internal voids was employed, allowing the flow of liquids and gases. This solution increases the sorption surface of the material, thereby enhancing the extraction efficiency. Each cured resin layer incorporates silica particles with a portion exposed to the surface, thereby providing accessible C18 functional groups for analyte interaction (Figure 1). Our prior research successfully extracted diazepam and medazepam from aqueous samples using a direct immersion solid-phase microextraction (SPME) approach. Building on this, we sought to exploit the volatility of PCBs by implementing a headspace extraction mode. This strategy is expected to minimize sample handling and improve selectivity by restricting analyte access to the sorbent to volatile compounds exclusively.

**Figure 2:**
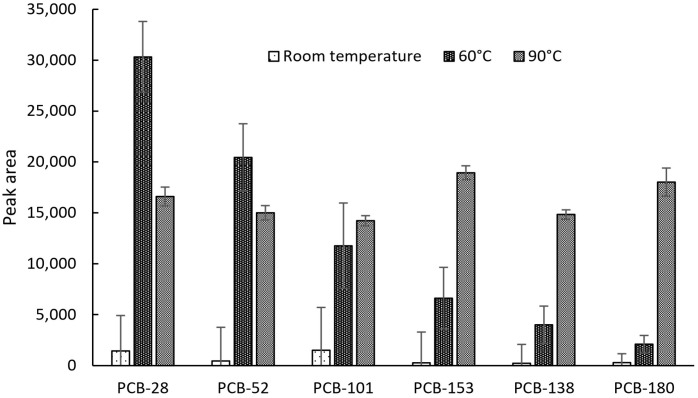
Impact of sorption temperature on extraction efficiency.

The extraction device was enclosed within a sealed extraction probe and subjected to temperature control to promote the volatilization of PCBs into the gas phase. The impact of the temperature on the effectiveness of analyte vapor extraction from urine enriched with six PCBs at a concentration of 5 ng/mL in a volume of 5 mL was investigated. The temperature values in this study were set to 20 °C, 60 °C, and 90 °C. The results presented in Figure 2 show a relation between sorption efficiency and temperature. At temperatures higher than 90 °C, leaks from the vials were observed, resulting in significant deviations in the results; hence, further experimentation with temperatures exceeding 90 °C was not attempted. Minimal extraction occurred at room temperature, while at 60 °C, high intensities were observed only for the least volatile PCBs and with relatively high RSD. At 90 °C, it was found that intensities were at similar levels regardless of analyte volatility, and RSD ranged from 3 to 7%. This experiment demonstrated that the sorption mechanism is temperature-dependent, improving the extraction efficiency of all six PCBs while simultaneously reducing RSD.

### 2.2. Optimization of the Extraction Procedure

**Figure 3:**
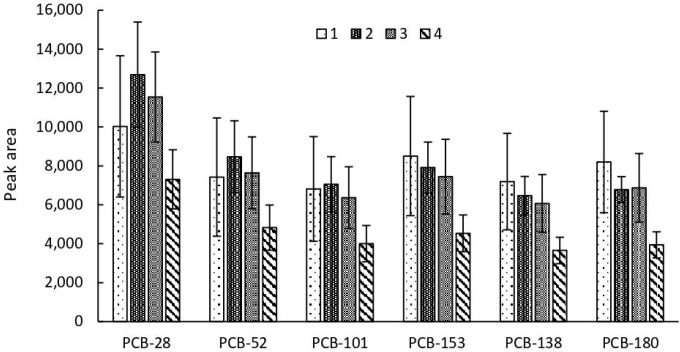
Impact of the number of extraction devices on extraction efficiency. Numbers 1–4 represent the number of sorbent devices used in the extraction process.

The influence of the number of sorbent devices on the extraction efficiency was also examined. Analyte vapor extraction from urine enriched with selected PCBs at a concentration of 5 ng/mL in a volume of 5 mL at the temperature of 90 °C was investigated. The results presented in Figure 3 indicate that the most favorable extraction conditions are obtained while using two sorbent devices, which translates into the highest extraction efficiency for half of the tested analytes and high repeatability values for all of the analytes. While sorbent quantity did not appear limiting, four devices yielded lower efficiency, likely due to suboptimal desorption conditions within the given solvent volume compared to fewer devices.

**Figure 4:**
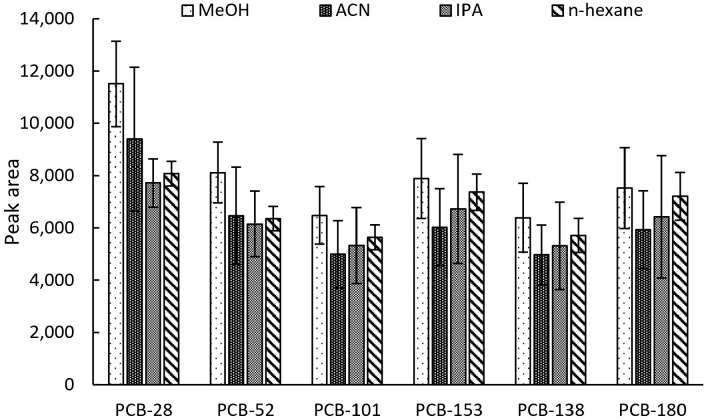
Impact of desorption solvent on extraction efficiency.

Results of the comparison of four desorption solvents: acetonitrile, methanol, hexane, and isopropanol have been presented in Figure 4. The highest peak areas were observed with the use of methanol for the desorption of the analytes from the 3D-printed extraction devices. However, the lowest RSD value was observed when using hexane. Methanol, a less environmentally harmful alternative to hexane, was selected in accordance with green chemistry principles. As such, further studies were conducted using methanol as the desorption solvent.

The kinetics of the sorption study (Figure S1) did not yield a definitive optimal sorption time, likely due to the varying volatilities of individual PCBs. Consequently, a sorption time of 60 min was selected as a practical compromise, sufficient for the majority of analytes. In contrast, desorption optimization (Figure S2) clearly indicated 20 min as the optimal duration, characterized by maximal analytical signal intensities and minimal relative standard deviation (RSD) values for most compounds.

### 2.3. Impact of C18 Functionalization on Extraction Performance

The experiment involved an extraction procedure using a sorbent device of the same geometry and size prepared without C18-modified silica as a control. While this sorbent exhibited some capacity for analyte extraction, it demonstrated significantly lower efficiency and reproducibility compared with the C18-modified device, as indicated by higher RSD values. These findings underscore the critical function of C18 functionalization in enhancing extraction performance and reliability. A comparative summary of extraction efficiency and RSD values for both device compositions is presented in Table 1.

### 2.4. Validation Study

To ensure the integrity of the analytical process, a negative control experiment was conducted using pure water as a sample matrix. The absence of any target analyte peaks confirmed the purity of both the self-fabricated sorbent and the overall analytical system.

The method’s linearity was assessed within a concentration range of 10 to 100 pg/mL. The calibration curves were linear in the corresponding ranges with a correlation coefficient higher than 0.957, as shown in Table 2. LLOQ of the analytical procedure is 10 pg/mL for all analytes.

The method’s precision and accuracy were evaluated by analyzing multiple quality control samples at each concentration level (15, 60, 90 pg/mL). The data for intra- and inter-day precision and accuracy from QC samples are summarized in Table 3, respectively. The results were within acceptable limits, showing satisfactory accuracy and precision of the assay.

### 2.5. Real Sample Analysis

The main objective of this study was to develop a precise method for the determination of PCBs in urine using 3D-printed extraction devices. Urine samples for the study were provided by the Division of Neonatology of the University Clinical Centre at Medical University of Gdańsk. Five urine samples collected from newborns were analyzed. In most of them, the presence of PCBs was observed, but their concentrations varied significantly between individuals. The lowest concentration among all analytes was observed for PCB-52, where the concentration could only be determined in two cases. A similar situation was noted for PCB-180, where the concentration could be determined for only one patient. The representation of the concentration level for each analyte found in urine is presented in Table 4.

PCBs, which were once widely used in various industrial applications, have been found to pose significant health risks due to their persistence and ability to accumulate in the environment. The concentrations varied among individuals, highlighting the importance of monitoring exposure towards PCBs. The presence of PCBs in newborns’ urine is particularly concerning, as their bodies have not been exposed to the environment in the same way as adults. The main source of exposure is most likely their mother’s milk and the presence of PCBs in the placenta, via which nutrients and gases are delivered to the fetus during early development [32,33]. This underscores the critical need for thorough monitoring of PCB levels in the environment and food web to protect the health of both mothers and their children. Furthermore, detecting PCBs in this population additionally emphasizes the urgent need for effective health interventions. Newborns are particularly sensitive to toxic substances due to their developing systems and lower body weight, making them more susceptible to adverse health effects. The fact that PCBs are present in newborns, who theoretically should be the most protected, indicates widespread contamination of our ecosystems and food webs. It is noteworthy that PCB levels detected in newborn urine were significantly lower (pg/mL) than those reported for adults (ng/mL) [18]. This contamination not only threatens human health but also disrupts ecological balance, affecting wildlife and natural resources. Importantly, their detection does not necessarily imply the occurrence of adverse health effects. Therefore, advancing our understanding and methods of detecting PCBs is essential for developing comprehensive strategies to mitigate their impact, ensuring both environmental and public health safety.

## 3. Materials and Methods

### 3.1. Chemicals

PCBs mixture: 2,4,4′-trichlorobiphenyl (PCB-28), 2,3,3′,5′-tetrachlorobiphenyl (PCB-58), 2,2′,4,5,5′-pentachlorobiphenyl (PCB-101), 2,2′,4,4′,5,5′-hexachlorobiphenyl (PCB-153), 2,2′,3,4,4′,5′-hexachlorobiphenyl (PCB-138), and 2,2′,3,4,4′,5,5′-heptachlorobiphenyl (PCB-180), each at 10 µg/mL dissolved in isooctane, was purchased from VWR International (Radnor, PA, USA). Deuterated internal standard 2′-chloro-2,3,4,5,6-pentadeuterio-1,1′-biphenyl (PCB-1) at 10 µg/mL dissolved in isooctane was purchased from Laboratory of the Government Chemist Ltd. (Teddington, UK). Acetonitrile (ACN), methanol (MeOH), isopropanol (IPA), and hexane (HEX) with GC–MS purity were provided by Merck (Darmstadt, Germany). Octadecyl-functionalized silica gel of undefined particle diameter (9–13% carbon loading; product No.: 553522; LOT: MKCQ2192) was purchased from Merck (Darmstadt, Germany). Anycubic Plant Clear resin was purchased from Anycubic (Shenzhen, China).

### 3.2. Samples

For a given study, urine samples were collected from newborns at the Division of Neonatology, University Clinical Centre at the Medical University of Gdańsk. There were five urine samples collected in test tubes. All samples were stored in the dark at a temperature of −80 °C until analysis. They were thawed directly before analysis to maintain sample integrity. 

### 3.3. 3D Printing Procedure—Fabrication of a Sorbent Device

The method for the fabrication of the extraction device was reported in our previous work [32]. Suspension of the sorbent particles in the resin was prepared in a two-stage process. First, C18 silica was weighed into a 50 mL falcon tube(the amount of C18-modified silica was 5 g for a 10% *w*/*w* suspension). Then, 20 g of photocurable resin was added and stirred using a glass rod to create a homogeneous suspension core. This core was afterwards diluted with more resin to finally achieve a homogenous batch of 50 g silica/resin suspension. Before each printing, a fresh batch of suspension was prepared.

**Figure 5:**
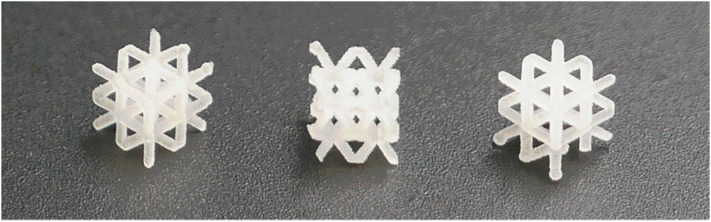
Photograph of 3D-printed extraction devices.

Presented volumetric cubic lattices (5 × 5 × 5 mm) were created using the X-cell shape with a side of 5 mm and an infill percentage of 30%. The shape can be used as a single piece or in multiples (Figure 5).

The extraction devices were developed using the Anycubic Photon Ultra Mono 2 (Anycubic, Shenzhen, China) and were conducted using the following parameters: Layer height was set to 100 µm, and bottom layer exposure time was set to 70 s, with 6 bottom layers. After the bottom layers, the exposure time of one layer was set to 15 s, and a 1 s light-off delay. The total printing time required was approximately 43 min, regardless of the number of printed devices.

After the printing process was completed, the uncured resin covering the solidified device had to be removed. For this purpose, printed devices were submerged in an Anycubic Wash & Cure machine (Anycubic, Shenzhen, China) filled with isopropyl alcohol, equipped with a magnetic stirrer for washing the prints and a UV light strip to cure them afterwards. After 6 min of rinsing, supports were removed from the printed devices, and prints were subjected to 6 min of UV light to ensure the completion of the curing process. The average mass of dry extraction devices was 34.74 mg (RSD = 2%, *n* = 10).

### 3.4. Primary Investigation of Sorption Properties Under Different Conditions

The printed and cured volumetric lattice-shaped devices underwent a study aimed at confirming their sorption properties in analyte vapors. For this purpose, extraction was carried out at 3 different temperature levels. Furthermore, to investigate the potential sorption properties of the resin itself, a negative control was conducted utilizing sorbent devices without silica.

### 3.5. Sample Preparation Procedure—Final Conditions

**Figure 6:**
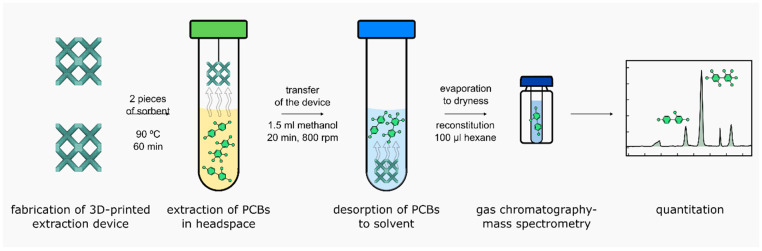
The procedure for sample preparation.

Two pieces of extraction devices were strung on a string and then soaked in methanol for 5 min to activate C18 chains bonded to silica particles. In a 12 mL glass vial, 5 mL of urine and 10 µL of deuterated internal standard at a concentration of 1 µg/mL were added. The devices were suspended in the vial so that they did not come into contact with the urine, and then the vials were closed and sealed with Parafilm. The vials were placed in an oven at 90 °C for one hour without agitation. After one hour, the vials were cooled down for 10 min. Then, the devices were transferred to a clean vial and submerged in 1.5 mL of methanol. A laboratory shaker was used to carry out the desorption procedure. Test tubes were placed for 20 min with shaking at 800 rpm. After this time, the printed extraction devices were removed from the solution. The next step was to evaporate the extract to dryness under vacuum at 22 °C (Labconco, CentriVap, Kansas City, MO, USA). Next, 100 µL of hexane was added to dissolve the extract, and the sample was briefly vortexed. Afterwards, the sample was transferred to a GC vial and analyzed directly (Figure 6). Printed extraction devices were designed for single-use applications. No studies have been conducted on their reuse during extraction processes.

### 3.6. Optimization of the Sample Preparation Procedure

The process of selecting the optimal desorption solvent for the presented procedure was based on comparing extraction efficiencies achieved using acetonitrile, methanol, hexane, and isopropanol as typical desorption solvents used with C18 sorbent powder. The sample from the sorption step contained all 6 PCBs in 5 mL of urine at a concentration of 5 ng/mL. After performing the extraction procedure for 60 min of sorption and 30 min of desorption, the desorption samples were subjected to analysis using GC-MS, and the results were used to calculate the extraction efficiency. To optimize the number of printed extraction devices used in the protocol, 4 sets containing 1, 2, 3, and 4 devices suspended in a tube were used. The experiment was performed according to the regular extraction protocol. All studies were conducted in three replicates to allow the calculation of the relative standard deviation.

Sorption kinetics were evaluated by performing a regular extraction protocol with varying sorption times (from 10 to 60 min, with 10 min intervals) and a fixed desorption time and plotting peak areas from desorbed samples afterwards. For the estimation of desorption kinetics, the same approach has been applied, with the only difference being that sorption time was fixed (on the value designated as optimal in the previous step) with a time range from 5 to 25 min (with 5 min intervals). All studies were performed in triplicates to enable relative standard deviation calculation.

### 3.7. Gas Chromatography–Mass Spectrometry

The chromatographic analysis of the extracts was performed using the GC-MS technique. An Agilent Technologies 7820A gas chromatograph and an Agilent Technologies 5977E MSD mass spectrometer were used. The chromatographic separation of the tested compounds was achieved using a 20 m long capillary column DB-8270D (Agilent J&W GC Columns, Santa Clara, CA, USA), made of fused silica, with an internal diameter of 0.180 mm and a film thickness of 0.36 µm. The above-mentioned column can operate in the temperature range of 60–325/350 °C.

The splitless mode of dosing was applied at a temperature of 320 °C, and the pressure value was 33.383 psi. The carrier gas was helium, and its flow rate was 1.3314 mL/min. The syringe of the dispenser was rinsed with hexane (100%) six times before dosing and six times after dosing the sample. The sample was dosed in a volume of 2 µL. A GC-MS transfer line temperature of 320 °C, an ion source temperature of 250 °C, and a quadrupole temperature of 180 °C were used. The technique of single ion monitoring (SIM) was applied. For each analyte, one ion was selected for quantitative analysis, and an additional two signals were chosen for identification purposes (Table 5).

The column temperature was initially held at 120 °C for 0.5 min, raised to 200 °C at the rate of 8 °C/min, and kept for 0.5 min. Then, the temperature was increased to 270 °C at a rate of 10 °C/min. From 270 °C the temperature rose to 320 °C at a rate of 12 °C/min and was maintained for 1 min. The post-run time was 3 min. The total analysis time was 23.17 min.

A solvent delay was fixed at 4 min, and the analytes were divided into 4 separate time segments: 4.00–13.50 min for PCB-28 and PCB-58, 13.50–15.00 min for PCB-101, 15.00–17.00 min for PCB-138 and PCB-153, and 17.00–23.17 for PCB-180.

### 3.8. Validation Study

#### 3.8.1. Calibration Curve and Range

The linearity of the method was assessed at a range between 10 and 100 pg/mL. Linearity was determined through the extraction of urine spiked at 7 concentrations: 10, 20, 25, 35, 50, 75, and 100 pg/mL with the use of printed extraction devices. The correlation coefficient consistently surpassed the threshold of 0.956.

#### 3.8.2. The Lower Limit of Quantitation

In the presented study, the lower limit of quantitation (LLOQ) was used as the criterion for assessing the range of analytical concentrations. Choosing LLOQ instead of LOQ allowed for a more reliable determination of the detection limit, which is crucial in the analysis of samples with very low analytical concentrations, where even minimal changes might have significant analytical implications.

#### 3.8.3. Precision and Accuracy

The precision and accuracy were calculated based on three concentrations: 15, 60, and 90 pg/mL. The determination of these parameters was divided into three days. Accuracy and precision assessment was performed by adding a specific amount of PCBs’ standards to pooled urine. For each concentration, the experiment was performed in three replications.

## 4. Conclusions

The study provides comprehensive insights into the extraction and analysis of selected polychlorinated biphenyls (PCBs) from urine samples using 3D-printed extraction devices. PCBs, which were once widely used in various industrial applications, have been found to pose significant health risks due to their persistence and ability to accumulate in the environment. The new sample preparation procedure was successfully developed and validated. The use of 3D-printed sorbent devices offers significant advantages compared to traditional solid-phase extraction techniques such as columns or dispersive extraction. The most important advantage is the ability to customize the shapes according to experimental needs. Furthermore, the use of 3D printing techniques significantly reduces the cost of sample preparation. The biggest cost is the purchase of C18 silica, which determines the main expense. The cost of printing one extraction device containing C18 silica is $0.40. In comparison, the cost of printing the same device without silica C18 is $0.02. Due to the analysis of trace amounts of PCBs in urine and the potential for contamination, the reuse of the extraction device was not attempted. In the case of vapor-phase extraction of analytes, it would be technically much more challenging to use non-immobilized sorbents or commercially available SPE columns. Furthermore, 3D printing technology allows for rapid prototyping and easy modification of the design, which are more difficult to achieve with traditional extraction methods. It is worth mentioning that the negative control has proven that the use of 3D-printed devices does not lead to contamination of the samples during the extraction procedure. The optimized method offers a promising approach for sensitive and environmentally friendly analysis of PCBs, contributing to efforts aimed at mitigating the health risks associated with PCB exposure. The development and application of new analytical methods can also lead to a better understanding of the dynamics of PCB transport and accumulation.

## Figures and Tables

**Table 1:** Comparison of the extraction efficiency and RSD for devices with and without C18-modified silica particles [*n* = 3].

	Extraction Devices Without C18 Particles		Extraction Devices with C18 Particles	
Analyte	Extraction Efficiency [%]	RSD [%]	Extraction Efficiency [%]	RSD [%]
PCB-28	24	12	31	5
PCB-52	30	12	35	3
PCB-101	36	13	40	2
PCB-138	31	32	43	8
PCB-153	32	27	41	8
PCB-180	28	55	43	10

**Table 2:** Parameters of calibration curves for each analyte [*n* = 4].

Analyte	Calibration Curve Regression Parameters		
	a	B	R^2^
PCB-28	0.0368	0.00212	0.969
PCB-52	0.2130	0.0429	0.989
PCB-101	0.0179	6.769 × 10^−4^	0.961
PCB-138	0.0118	4.943 × 10^−4^	0.975
PCB-153	0.0138	7.003 × 10^−4^	0.957
PCB-180	0.0704	0.00692	0.989

**Table 3:** Intra- and inter-day accuracy and precision calculated for each analyte. The accuracy is expressed as a percent of the nominal concentration value, whereas precision is expressed as relative standard deviation.

	Accuracy						Precision					
	Intra-Day [%] (*n* = 3)			Inter-Day [%] (*n* = 9)			Intra-Day [%] (*n* = 3)			Inter-Day [%] (*n* = 9)		
Concentration [pg/mL]	15	60	90	15	60	90	15	60	90	15	60	90
PCB-28	105	98	103	109	104	104	11	7	5	14	9	7
PCB-52	97	102	107	103	103	109	8	5	1	11	9	2
PCB-101	110	105	105	114	108	106	10	7	4	12	8	5
PCB-138	107	103	102	112	107	106	7	7	3	9	9	5
PCB-153	109	104	102	115	108	105	11	8	5	14	8	6
PCB-180	109	100	97	111	104	96	8	8	5	14	9	7

**Table 4:** PCB concentrations (pg/mL) in urine samples from five newborns.

Patient Number	PCB-28 [pg/mL]	PCB-52 [pg/mL]	PCB-101 [pg/mL]	PCB-138 [pg/mL]	PCB-153 [pg/mLl]	PCB-180 [pg/mL]
1	43.01	<LLOQ	50.36	73.15	65.71	<LLOQ
2	29.90	10.81	33.31	41.39	40.51	<LLOQ
3	31.16	<LLOQ	35.88	52.78	46.79	<LLOQ
4	33.35	<LLOQ	36.94	51.01	53.07	<LLOQ
5	29.62	<LLOQ	30.79	41.35	37.37	<LLOQ

**Table 5:** Summary of data for analytes and internal standard.

Analyte	Abbreviation	Retention Time [min]	Quantifier Ion [m/z]	Qualifier Ions [m/z]	Boiling Point [°C]
2,4,4′-trichlorobiphenyl	PCB-28	12.14	256	186; 150	334.36
2,3,3′,5′-tetrachlorobiphenyl	PCB-58	12.73	292	186; 150	374.95
2,2′,4,5,5′-pentachlorobiphenyl	PCB-101	14.95	326	128; 254	412.3
2,2′,4,4′,5,5′-hexachlorobiphenyl	PCB-153	16.93	360	145; 290	446.99
2,2′,3,4,4′,5′-hexachlorobiphenyl	PCB-138	17.23	360	290; 145	446
2,2′,3,4,4′,5,5′-heptachlorobiphenyl	PCB-180	22.39	394	252; 324	479
2′-chloro-2,3,4,5,6-pentadeuterio-1,1′-biphenyl (internal standard)	PCB-1	6.48	193	195; 157	

## Data Availability

Data is contained within the article and supplementary material.

## Supplementary Materials

The following supporting information can be downloaded at https://www.mdpi.com/article/10.3390/ijms26062755/s1.
