# Supplementary material for: Headspace Extraction onto a 3D-Printed Device for GC-MS Quantification of Polychlorinated Biphenyls in Newborn Urine
Source: Int J Mol Sci. 2025 Mar 19;26(6):2755. doi: 10.3390/ijms26062755 (PMC11942790; doi:10.3390/ijms26062755)
Supplement: Supplementary file 1 [file ijms-26-02755-s001.zip › ijms-3508730-supplementary.pdf]

Supplementary Materials

Headspace Extraction onto a 3D-Printed Device for GC-MS Quantification of Polychlorinated Biphenyls in Newborn Urine

Authors:

Paweł Georgiev[a], Szymon Ulenberg [a], Dagmara Kroll[a], Bartosz Marciniak[a], Izabela Drążkowska[b], Tomasz Bączek[a], Justyna Płotka-Wasylka[c,d], Mariusz Belka[a,*]

Affiliations:

[a] Department of Pharmaceutical Chemistry, Medical University of Gdańsk, J. Hallera 107, 80-416, Gdańsk, Poland

[b]Division of Neonatology, Medical University of Gdańsk, 80-210 Gdańsk, Poland

[c] Department of Analytical Chemistry, Faculty of Chemistry, Gdańsk University of Technology, G. Narutowicza 11/12, 80-233 Gdańsk, Poland

[d]BioTechMed Center, Gdańsk University of Technology, G. Narutowicza 11/12, 80-233 Gdańsk, Poland

Corresponding author: Mariusz Belka, mariusz.belka@gumed.edu.pl

Contents:

Figure S1. Sorption kinetics plot for studied analytes.

Figure S2 . Desorption kinetics plot for studied analytes.

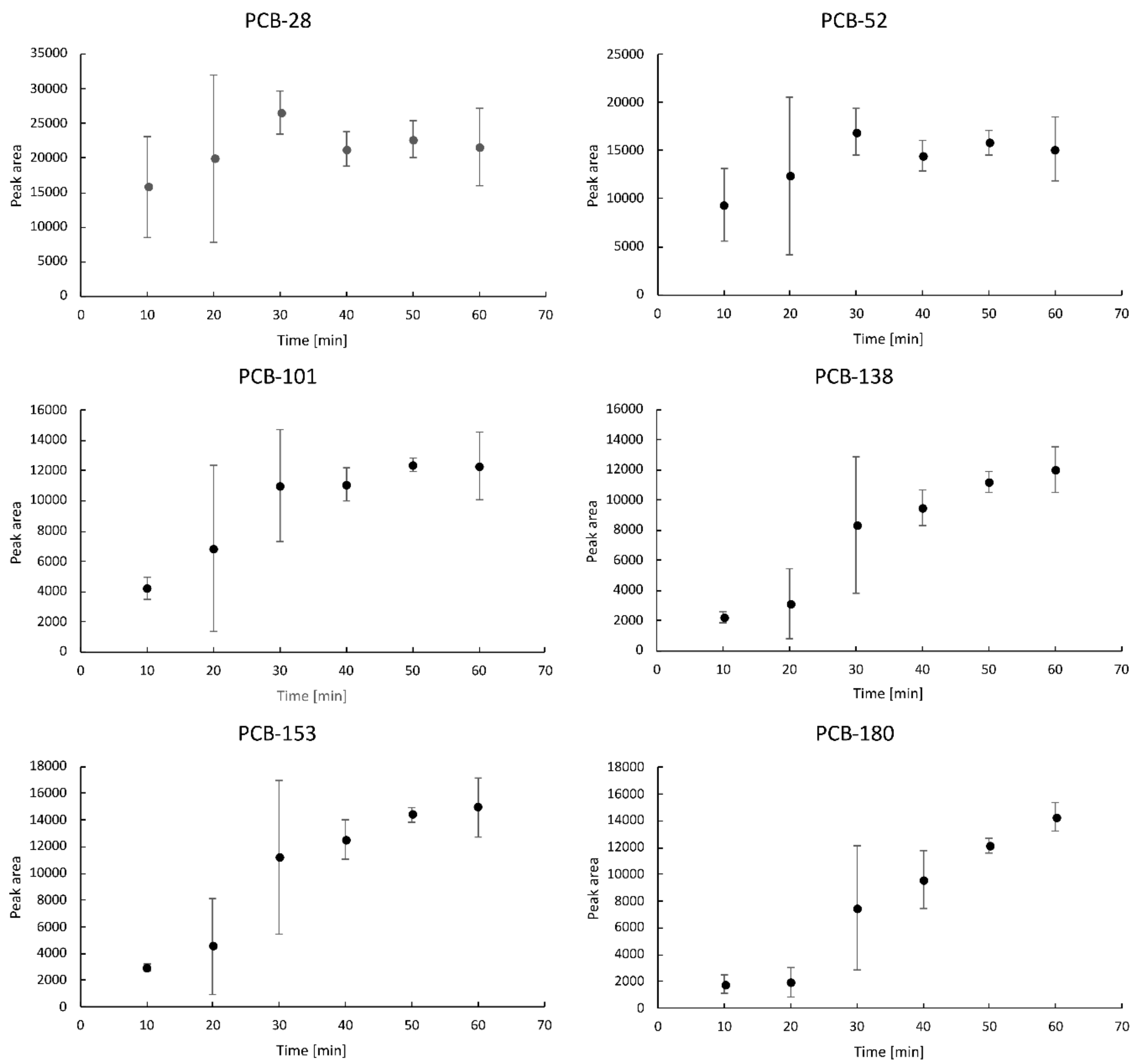

Figure S1. Sorption kinetics plot for studied analytes.

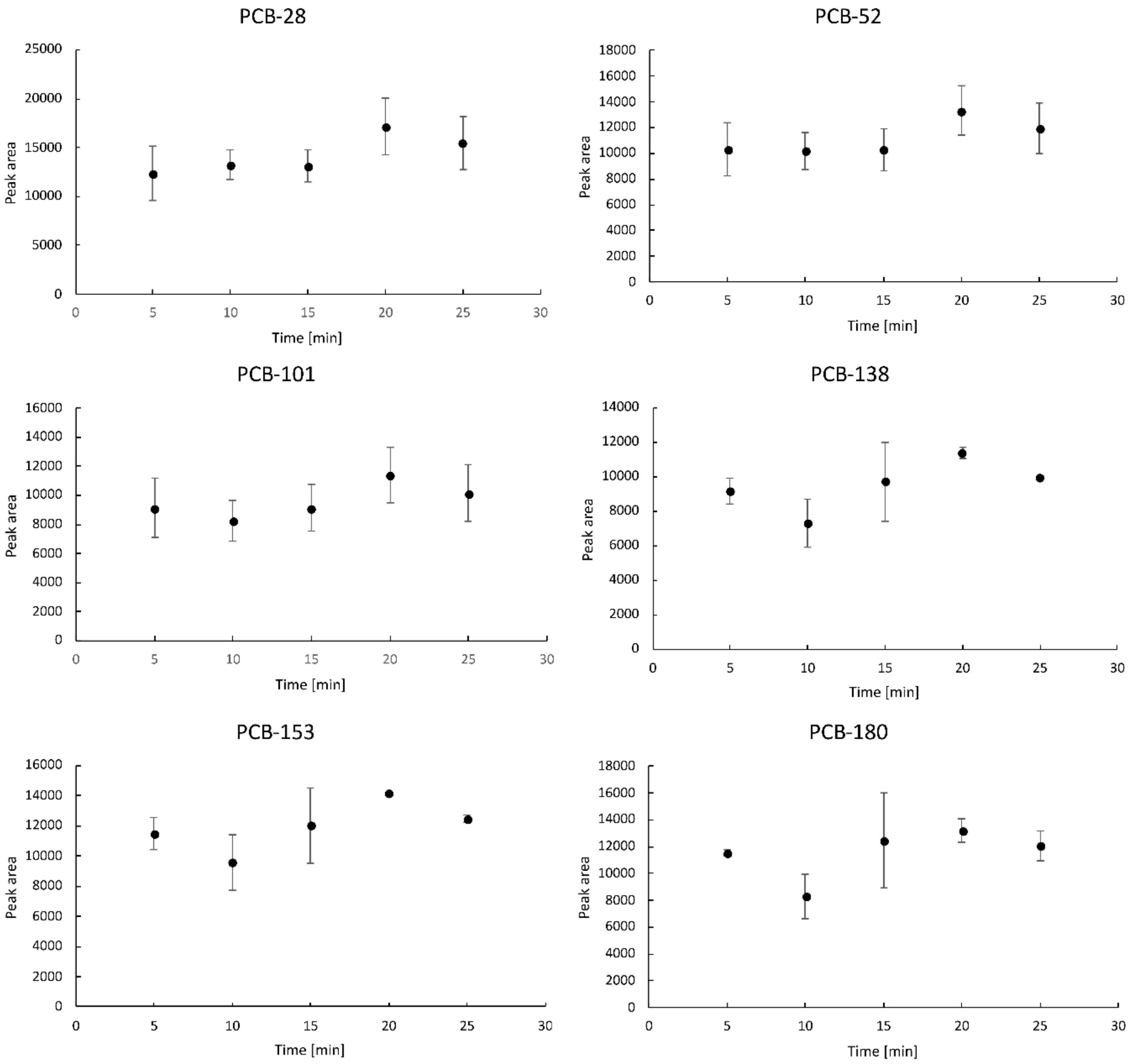

Figure S2 . Desorption kinetics plot for studied analytes.
